# A “Community Fit” Community-Based Participatory Research Program for Family Health, Happiness, and Harmony: Design and Implementation

**DOI:** 10.2196/resprot.4369

**Published:** 2015-10-28

**Authors:** Cissy SS Soong, Man Ping Wang, Moses Mui, Kasisomayajula Viswanath, Tai Hing Lam, Sophia SC Chan

**Affiliations:** ^1^ School of Public Health University of Hong Kong Hong Kong China (Hong Kong); ^2^ School of Nursing University of Hong Kong Hong Kong China (Hong Kong); ^3^ Family and Community Service Hong Kong Council of Social Service Hong Kong China (Hong Kong); ^4^ School of Public Health Department of Social and Behavioral Sciences Harvard University Boston, MA United States

**Keywords:** communication, community-based participatory research, family, happiness, harmony, health

## Abstract

**Background:**

A principal factor in maintaining positive family functioning and well-being, family communication time is decreasing in modern societies such as Hong Kong, where long working hours and indulgent use of information technology are typical.

**Objective:**

The objective of this paper is to describe an innovative study protocol, “Happy Family Kitchen,” under the project, “FAMILY: A Jockey Club Initiative for a Harmonious Society,” aimed at improving family health, happiness, and harmony (3Hs) through enhancement of family communication.

**Methods:**

This study employed the community-based participatory research (CBPR) approach, and adopted 5 principles of positive psychology and the traditional Chinese concepts of cooking and dining, as a means to connect family members to promote family health, happiness, and harmony (3Hs).

**Results:**

In-depth collaboration took place between an academic institution and a large nongovernmental community organization association (NGO association) with 400 social service agency members. Both groups were deeply involved in the project design, implementation, and evaluation of 23 community-based interventions. From 612 families with 1419 individuals’ findings, significant increases in mean communication time per week (from 153.44 to 170.31 minutes, *P*=.002) at 6 weeks after the intervention and mean communication scores (from 67.18 to 69.56 out of 100, *P*<.001) at 12 weeks after the intervention were shown. Significant enhancements were also found for mean happiness scores 12 weeks after the intervention (from 7.80 to 7.82 out of 10, *P*<.001), and mean health scores (from 7.70 to 7.73 out of 10, *P*<.001) and mean harmony scores (from 7.70 to 8.07 out of 10, *P*<.001) 6 weeks after the intervention.

**Conclusions:**

This was the first CBPR study in a Hong Kong Chinese community. The results should be useful in informing collaborative intervention programs and engaging public health researchers and community social service providers, major stakeholders, and community participants in the promotion of family well-being. Furthermore, this study has generated an effective practice model for the improvement of family communication and well-being. Challenges in maintaining research rigor during data collection and program implementation were observed, and should be considered during future program planning.

## Introduction

### Family Communication

Communication has been identified as a core factor leading to family harmony and well-being. Inadequate communication may lead to verbal abuse, neglect, and indifferent relationships within the family, thus exerting negative effects on health, happiness, and harmony (the “3Hs”) [1-2]. Effective communication—in the form of interaction through quality family time and direct verbal contact between family members—is associated with better mutual acceptance, conflict reduction, and harmonious family relationships [3].

Although Chinese individuals place great importance on family, certain changes in family structure, such as cross-border families in which parents must work outside Hong Kong, role changes such as grandparents becoming child caretakers, and excessively busy lifestyles, have led to inadequate time for and quality of family communication. [1] A recent local survey [4] showed that nearly 50% of respondents had never or only occasionally listened to and heeded their parents’ advice on important matters. Similarly, over half rarely or never communicated with siblings on such matters. Our own study on family and health information trends [5] showed that half of the respondents reported a knowledge deficit in communicating positively with their family members. Therefore, interventions to improve family communication are warranted.

### Community-Based Participatory Research

Community-based participatory research (CBPR) is an approach that combines both research methods and community capacity-building strategies to bridge the gap between knowledge produced through research and actual interventions and policies [6,7]. The approach is particularly attractive to academics and public health professionals struggling to address persistent problems of health care disparities in different populations [8], with the aim of combining knowledge and action for social change to improve community health and reduce inequality. CBPR is increasingly used to promote family well-being, with most studies conducted in Western populations, which have a family culture distinct from that of the Chinese.

### Positive Psychology

Several studies have demonstrated the benefits of applying the principles of positive psychology to family well-being [9-12]. The principles of positive psychology are instrumental in the conceptualization of services provided to parents, family members, teachers, and other adults with children [13]. “Cooking” and “dining” form an easily achievable and well-accepted channel of communication between family members. Dining with family is valued by the Chinese as an important event, in which communication is emphasized in traditional culture, focusing on the individual’s obligation to maintain family well-being. As part of the project, “FAMILY: A Jockey Club Initiative for a Harmonious Society,” we combined these concepts to develop a CBPR project entitled “Happy Family Kitchen” (HFK), with a framework of 5 themes: praise and gratitude, flow, happiness, health, and savoring. The application of these elements in daily life is expected to further promote family health, happiness, and harmony (3Hs) by enhancing communication among family members more practically. Specifically, we aimed to build effective community partnerships with various stakeholders and NGOs, to establish a useful CBPR model based on positive psychology for promoting family well-being.

### Objective

The main objective of this paper is to describe the project protocol entitled “Happy Family Kitchen,” involving collaboration and partnership between academia and community stakeholders, using a community-based participatory approach, and having an implementation target of enhanced family functioning and communication to promote health, happiness, and harmony (3Hs). The effectiveness of a series of minimal preventive interventions in terms of structure, process, outcomes, and impact was also evaluated.

## Methods

The project was planned and implemented according to the guidelines developed by Stith [14] and described as follows.

### Ensure the Community is “Ready”

Community readiness is an important factor in enhancing community cooperation, participation, and resource sharing, and in maintaining the intracommunity climate and sustainability to implement prevention programs effectively [15-17]. Sufficient community capacity, recognition of existing problems, sufficient programs, and identification of key champions are essential indicators of community readiness [17-20]. Several in-depth interviews with community leaders were conducted in 2011 by the principal investigator to gain a better understanding of community needs regarding issues affecting family health, happiness, and harmony (3Hs) in Hong Kong [1]. Findings supported a need in the community to design effective education campaigns to further promote family communication to improve the 3Hs.

### Develop “Community Coalitions”

Effective community coalitions facilitate good internal project functioning through an inclusive climate and mutual trust among community partners [11,13]. The HFK project collaborated with a major NGO, the Hong Kong Council of Social Service (HKCSS), which is an umbrella social service organization with more than 300 member agencies. The HKCSS was involved in recruitment, motivation of their member agencies to participate, and the design, implementation, evaluation, and dissemination of results. A steering committee including the HKCSS, governmental department, representatives of different nongovernmental organizations, and different community stakeholders was formed. Various components of project design, structure, means of implementation, outcome focus, and evaluation method were discussed throughout the project.

### Confirm the Project Fits the Community

Community fit is achieved by meeting the identified needs of the community and designing appropriate interventions for targeted cultural groups. Programs that fit the community tend to be flexible, responsive, cost-effective, and culturally appropriate [21,22]. In exploring the community context, risk factors related to problems and current limitations should be explored before any intervention takes place; such community fitness information was examined prior to the start of the HFK project.

### Program Fidelity

Program fidelity refers to the full extent of an intervention program being delivered as planned, and is associated with positive program outcomes [16,21,23,24]. Strategies to enhance fidelity include adequate staff training [25], setting minimum dosage and adherence levels [24], and providing ongoing supervision and feedback to involved parties [16,25]. These were adopted in our projects as follows:

### Training for Program Leaders

To strengthen the program’s fidelity, a 2-day “train-the-trainers” workshop was organized to equip NGO social workers with essential knowledge and skills to design and implement intervention programs for families. After this training, social workers designed their own programs focusing on 1 of 5 themes chosen by them with the same primary aim to improve family communication, using family cooking or dining as a platform. Each activity-based program consisted of 1-2 sessions of at least 2 hours each concerned with the core content, and 1 shorter booster session. Program content was designed according to principles of the chosen theme and characteristics and needs of the specific participants or users (eg, families with school-age children, elderly members, or disabled persons).

A set of requirements for the specific program was developed and monitored by the project steering committee as follows. The target population was to be not less than 50 families with at least two members per family in each activity. The program structure was to consist of 1 or 2 core intervention sessions and 1 booster session 6 weeks later. The theme was to be 1 of 5 designated themes of positive psychology, to be chosen by the social workers of each service agency. Finally, the program was to be delivered by trained social workers.

### Process Evaluation

Given its importance as a component of a comprehensive assessment [7], a detailed process evaluation was conducted on our project to assess the quality and fidelity of the intervention delivered and the acceptability and feasibility of the programs, and to identify areas for improvement. Comprehensive evaluation tools were used and included a standardized program observation form, participant questionnaire evaluation, attendance records, and reports from the HKCSS and participating social service agencies.

A standardized program observation form was developed to monitor activities by 2 observers from Hong Kong University (HKU) and 2 from the HKCSS. The form assessed content fidelity, participant levels of interest, and program involvement, program interactivity level of delivery methods, strategies to enhance motivation of participants, time management, program preparation and elements, resources, success of implementation, implementation quality, degree of achievement of overall objectives, barriers and difficulties encountered during the program, and manpower allocation.

Behavioral checklists and program rundowns were used to triangulate findings on program fidelity. NGO agency workers addressed checklists by identifying methods as to how programs could deliver messages and encourage desired behaviors. The content and rundown of each program were designed by individual agencies and monitored by the HKCSS and HKU. Both behavioral checklists and rundowns were used to assess program adherence, content, and doses delivered.

### Provide Adequate Resources, Training, Technical Assistance, and Evaluation

Successful prevention programs require sufficient resources, which include adequate and reliable funding, stable staffing and organization, adequate training, technical assistance, and program evaluation [26-28]. Evaluation is an important element in judging the impact of a program on the targeted community and its people [27-29]. Our project fulfilled these requirements because it is a part of the larger project “FAMILY: A Jockey Club Initiative for a Harmonious Society” (FAMILY Project) funded by the Hong Kong Jockey Club Charities Trust. Adequate and reliable funding from the FAMILY project and the HKCSS provided stable and consistent staffing. A registered social worker was recruited as the project manager for project planning, initiation, and implementation throughout the project. The HKCSS also provided sufficient administrative support and technical assistance. A detailed evaluation plan, led by the University of Hong Kong (HKU), was incorporated at an early stage, which helped to define the specific objectives and outcomes. The evaluation or assessment tools, with measureable outcomes, also served to guide the design of the interventions. The research team at HKU was also funded by the FAMILY project.

### Ethical Statement

Ethical approval was granted by the Institutional Review Board (IRB) of the University of Hong Kong/Hospital Authority Hong Kong West Cluster. Written informed consent was obtained and recoded verbatim, and the procedure was approved by the IRB.

## Results

### Ensure the Community is “Ready”

Findings from our earlier qualitative study [1] suggested that communication and family interaction were core components contributing to the family 3Hs. Families of lower socioeconomic status (SES) had fewer financial, human, and social resources. Providing tangible support (eg, educational opportunities, sufficient childcare facilities, vocational skill training) was perceived as the most effective way to improve family well-being. Hence, the HFK focused on low SES families.

A needs assessment was conducted with the local community stakeholders prior to project implementation in the low SES district of Yuen Long. The results showed that the stakeholders recognized the problem but did not have any plan to organize related interventions. This suggested that the community was in a preplanning stage and further strategies to improve community readiness were needed before any specific prevention programs could be planned [19,30]. The findings of the needs assessments also helped to identify key local champions in family services and programs, and led us to seek their support to facilitate program adoption [23,25]. The stakeholders suggested that communication and family interaction needed to be improved for the promotion of family well-being. In addition, further public education and more community resources were needed, so that many stakeholders would be mobilized to collaborate.

The CBPR approach was adopted to collaborate with NGOs and community leaders to fill the gaps between community needs and social service provision. A positive climate of program implementation was maintained, which included the confirmation of rewards, support structures, and assistance in overcoming barriers and resistance [30]. This also helped to deepen collaboration among the main NGO and service units.

### Develop “Community Coalitions”

A steering committee including the HKCSS, representatives of different nongovernmental organizations, and different community stakeholders was formed. It also involved the governmental Social Welfare Department, which provided strong support for the community coalition’s leadership. Several task force and work-level meetings were organized during the project planning and implementation periods. Various components of project design, structure, means of implementation, outcome focus, and evaluation method were discussed throughout the project. The opinions of both stakeholders and academics were incorporated into the project. Strong support and assistance were provided by various stakeholders and partners throughout project implementation at both the organization and community levels. A total of 19 organizations with 23 service units joined the project, with an expanded network with other organizations and local government departments, including the local district service unit of the Social Welfare Department, schools, and a community health services provider.

### Confirm the Project Fits the Community

The needs assessment identified several risk factors threatening family well-being, including community factors that contributed to inadequate communication, such as geographical barriers, long working hours, and families with cross-border relations or different generations living together. We adopted simple, acceptable, and easily practiced interventions introducing positive attitudes and appropriate communication skills among family members. The idea of incorporating the notions of “kitchen,” “family cooking,” and “having meals together” was also well accepted by both stakeholders and NGO social workers.

We hypothesized that the family 3Hs would be improved by increasing family communication quality and time through the intervention programs. Specifically, the project was conducted in 3 phases:

#### Phase 1: Gestation

This phase included planning, implementing, and evaluating a series of events and programs to increase public awareness of this project and prepare social workers as interventionists. Ideas for and details of the implementation logistics of activities were identified, discussed, and carried out. These promotion and preparation activities included a training program for social workers to equip themselves with the knowledge and skills of positive psychology and the design of community-based programs on family dining and cooking. An inauguration event was held to promote the aims of this CBPR project and arouse community stakeholders’ and the public’s awareness of the HFK and the family 3Hs. A series of engaging activities including game booths, family workshops, and sharing by guests were held to enhance the culture of positive family communication among participants. Exhibitions and leaflet distribution were also conducted to introduce the HFK project and the concepts of family health, happiness, and harmony (3Hs).

#### Phase 2: Dissemination

To promote the family 3Hs, 21 community programs were designed, delivered, and evaluated using conceptual framework theory developed based on 5 themes from positive psychology, including praise and gratitude, flow, happiness, health, and savoring. These 5 themes were strategically selected based on their associations with family well-being, as follows.

“Praise and gratitude” involved the expression of thanks and an emotional sense of appreciation. Praising the strength and goodness of family members through words or actions can increase their satisfaction level and further motivate them toward positive behavior. Being recognized by family members can help reduce negative emotion, strengthen family relationships, and encourage family members to overcome obstacles [31].

“Flow” is a concentrated personal experience involving an ecstatic psychological state, achieved through the completion of a challenge using their own strength and skills, such as being focused on cooking/dining with family; people in this state are completely focused and feel that time passes quickly. Csikszentmihalyi [32] believed that the only way to take ownership of one’s life is by learning to direct energy and control attention. Enjoying a “flow” moment can help to increase the ability to face obstacles and provide a buffer against the effects of negative emotion. It also helps to improve attention, self-efficacy, and stamina.

“Happiness” is a positive emotion and psychological state. It can be expressed in terms of both cognitive and affective aspects. Types of happiness include short periods of pleasure/joyfulness resulting from external stimulation without thought or consideration, and longer periods of gratification/satisfaction triggered by doing something enjoyable. Practical behavior like sharing a happy experience with family members at mealtimes improved this emotion.

“Health” can be separated into 2 aspects: mental and physical. The former is focused on the development of positive thoughts and improved resilience, and the latter on a healthy diet and better nutrition. Enjoying fruits and vegetables with family members is suggested as a simple and practical start to promoting “health” at mealtimes.

“Savoring” is not just a technique of relaxation, but also an attitude toward life: living for the present moment. It combines observing mindfulness with nonjudgmental attitudes. To practice “savoring” at mealtimes, we recommend slowing down the pace of eating and treasuring good times when dining with family. This “savoring” can help to reduce stress and depression, and broaden observation and mindset to achieve goals and become more relaxed. Langer also found that it could improve positive emotion, creativity, and physical health [33-35].

**Figure 1:**
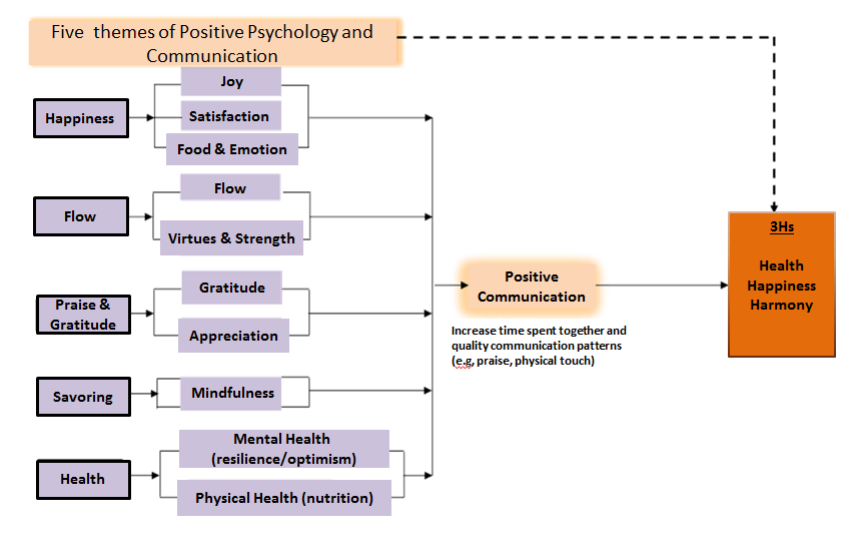
Conceptual framework of the Happy Family Kitchen intervention program.

These 5 themes were integrated into a conceptual framework for our community intervention programs, as shown in Figure 1. Throughout the thematic programs, participants would experience components of one of the themes: joyfulness and satisfaction in happiness, flow and ecstasy in flow, appreciation and thankfulness in gratitude, mindfulness in savoring, and resilience or a healthy diet in health. As community-based participatory and minimal approaches were adopted, interventionists used different means of delivery; however, only 1 of the 5 theme messages was included in their programs. The aim of this design was to simplify interventions and compare the effectiveness of themes in each intervention to identify the most effective and sustainable theme. It was believed that focused thematic programs would improve the sustainability of program impact; however, because the means of message delivery vary, intervention dose may be affected. Thus, intervention hours were controlled to ensure adequate dosage in the program. Certain behavioral indicators were suggested and it was hypothesized that changes in behaviors through the 5-theme program would improve positive communication within families, by increasing their communication time or patterns, and that such enhancement would lead to the hypothesized enhanced family 3Hs. Participants’ behavioral changes, family communication, and the 3Hs were evaluated both quantitatively and qualitatively.

#### Phase 3: Consolidation

This phase included the publication and release of a “Happy Family Cookbook” to promote the project’s theme and disseminate its methods and content. Finally, a practical knowledge-sharing forum was organized for social workers and other major stakeholders to showcase their programs through exhibits (including photos, feedback, and homework from participants) and discuss their experiences and the outcomes of the HFK project.

A series of territory-wide promotion and publicity strategies were implemented, including publication of the “Happy Family Cookbook” and “Practice Manual,” leaflets, street banners, a website, video promotions, newspaper and magazine articles, a radio program, and the distribution of souvenirs.

### Training, Proposals, and Specific Programs

#### Training of Social Workers

Before the intervention programs began, training sessions were delivered by a clinical psychologist and a nutritionist to introduce the theory and practice of positive psychology, and a healthy diet and nutrition, with 50 recorded attendees. HKU researchers explained scientific evaluation methods, including the principles of randomized controlled trials (RCTs). Training was interactive through the use of thematic games and group discussions. To boost the effects of this training, a set of manuals covering the content, together with proposal-writing guidelines, was distributed to participants. Moderate significant increases in perceived knowledge of positive psychology were observed immediately after training (*P*<.001) and were sustained at 6 months. Similar significant change was found for perceived ability to use positive psychology more frequently to advance family relationships and the 3Hs.

A total of 21 proposals from 23 units of 19 NGOs were submitted, received, revised, and vetted by the steering committee, which consisted of representatives of project organizers, academia, governmental departments, nongovernmental organizations, community stakeholders, and partners. The steering committee members provided valuable comments and suggestions regarding proposal designation and implementation and improved the intervention feasibility. Most (n=13) programs adopted gratitude as the theme; 3 adopted happiness, 2 were about health and 2 about flow, and only 1 was about savoring.

#### Fidelity Check

The results of a check on fidelity suggested that the community-based family programs were implemented successfully and well received by the participating families. Overall, high mean scores for program implementation (5.21, SD 1.15) and quality (5.10, SD 1.13) were rated by the observers on a Likert scale from 1 to 7. A large number of families/participants (1006 families with 2447 people) were recruited. Thematic positive psychology activities concerned with cooking and eating were attractive to many and the programs received favorable responses from almost all participants. Families demonstrated great interest in the activities, with a high level of participation, which was consistent with observers’ reports (mean 5.59, SD 0.93). Furthermore, many participants showed a positive response to homework assignments. However, from the observers’ view, the message delivered within programs was highly dependent on social workers’ level of understanding of the selected theme and their willingness to provide high-quality services. Comprehensive questionnaires at 4 separate time points were considered too time consuming and repetitive by participants.

#### Evaluation

The Yuen Long district (including Tin Shui Wai) was selected because of its unfavorable social and economic environment, in which there is a high demand for community services because of low socioeconomic status and a high prevalence of domestic violence. In 2010, newly reported battered spouse cases in Yuen Long accounted for 9.6% of all cases in Hong Kong [36]. Yuen Long also had low income in 2010; the proportion of households with a monthly household income of less than HKD $10,000 was 27% in Hong Kong, while for Yuen Long it was 30.4% [37].

**Figure 2:**
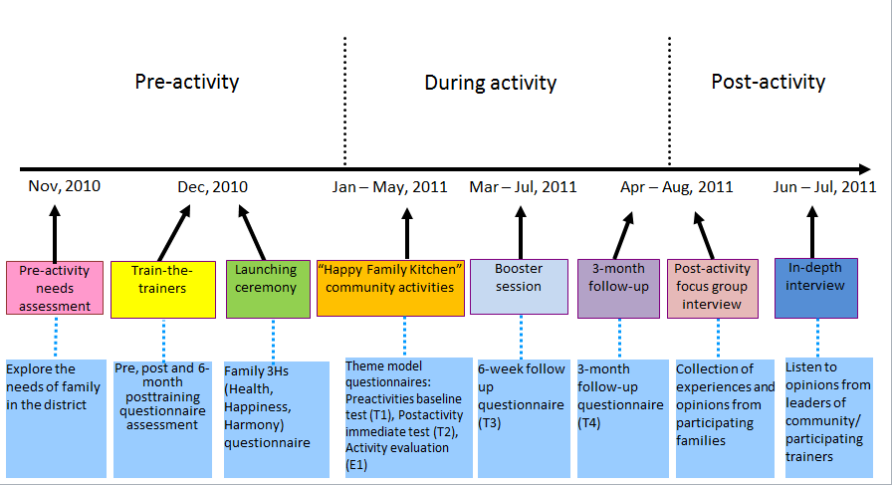
Evaluation of the Happy Family Kitchen Project.

Outcome changes in both participants and social workers were measured both quantitatively and qualitatively. For participants, measures of positive psychology behavioral changes, family communication, and the 3Hs were investigated. Using qualitative methods, more in-depth information was obtained, including feelings and satisfaction levels, specific examples of behavioral changes and interpersonal relationships, and longer-term impacts on families and professionals. Details of the evaluation framework are shown in Figure 2.

The project evaluation showed improvement in key outcomes, including awareness and behaviors in positive family communication and practices, and the family 3Hs. Among 1419 individuals from 612 families, mean communication time significantly increased from 153.44 to 170.31 minutes (*P*=.002 per week at 6 weeks after the intervention and the mean communication score increased from 67.18 to 69.56, *P*<.001, out of 100 at 12 weeks after the intervention) [38]. The overall mean happiness, health, and harmony scores (range 0-10, higher scores are better) increased from 7.80 (preintervention) to 7.82 (12 weeks after the intervention; *P*<.001), 7.70 to 7.73 (at 6 weeks; *P*<.001), and 7.93 (preintervention) to 8.07 (at 6 weeks; *P*<.001), respectively. These findings were supported by the qualitative interviews, which showed a high level of satisfaction among participants and social workers.

Qualitative data were examined by a panel of 2 researchers; transcripts were analyzed by thematic content analysis, following the guidelines recommended by Morse and Field [39]. The results from content analyses revealed that the intervention programs, regardless of the theme applied, were effective in improving family communication. Effectiveness varied from mild to intense; for example, awareness of the importance of family communication, mutual empathy, and reflections on the parent-child relationship.

We would chat more while having meals together…we had never thought about that in the past…In the past, just my daughter kept talking alone…Now, my husband would start talking…actively...

A mother, U6 16A Group 2

## Discussion

### Principal Findings

To the best of our knowledge, the Happy Family Kitchen project was the first CBPR in Asia to focus on promoting family communication and the 3Hs. By adopting a CBPR approach, we effectively engaged public health researchers, community service providers, major stakeholders, and local community residents in an active partnership. The project also demonstrated the feasibility of applying positive psychology in community interventions to promote the family 3Hs.

The evaluation results showed that participants generally had positive comments about the programs, in that they provided opportunities for their family members to gather and learn communication skills to improve the family 3Hs. The process evaluation also showed that the community programs were well received by participating families and appreciated by community stakeholders.

### Strengths

The project adopted a CBPR approach, which could be applied to diverse social service settings and target audiences. The project fulfilled community needs: the popular topic of family cooking and dining, suggested by the community as a method to promote the family 3Hs, and the adoption of a simple intervention design, which increased project acceptability and practicality for later sustainability in the community. The fidelity results suggested that the community-based family programs were well received by participating families, who demonstrated interest in the programs and a high level of participation. Participants also were positive in their reflective feedback. The project helped to build the capacity of a cluster of local social service units to promote the family 3Hs, and the rigorous program evaluations provided clear evidence of the effectiveness of the interventions. By engaging a large number of NGOs and service units, the project may also promote the adoption of the family 3Hs through social norms in Hong Kong.

### Limitations and Recommendations

#### Program Quality

As the interventions were developed by different NGOs, variations in the background of the NGOs, stakeholders, social workers, and other community members constituted a noticeable risk such that the dose, content delivery, and quality of the programs could vary. To increase the fidelity of program delivery, the project developed a theoretical framework to guide NGOs in design and implementation, and all program proposals were vetted by the project organizing committee to ensure they fit the theory and objectives. However, we found that a few interventionists mainly focused on positive psychology and communication without linking these to the family 3Hs.

This project was evaluated in detail, which may have created a burden for some community partners, in particular those without research experience. Future studies may consider more intensive training for such partners to build their research capacity. Communication and support should be provided between academic and community partners during the implementation process, to increase awareness of the importance of the project’s goals evaluation, and improve the quality of project implementation and future project planning.

#### Participant Recruitment and Retention

Community NGO partners typically focused on recruiting participants through their own networks, or their “comfort zone.” Although such an approach may be effective in terms of recruitment, it may limit generalizability. Many people who need the service may not know how to find it. It is important to encourage community partners to widen their “comfort zone” and proactively recruit individuals in need of these programs, for example, those of low socioeconomic status or male participants (who are rare in CBPR programs). To extend the project’s reach, different and more aggressive recruitment methods were proposed and adopted, such as door-to–door visits, street booths to communicate with the target audience, publicity work at community events, and use of the media to influence the community. However, a high attrition rate resulting from recruiting those not interested or too busy would lead to a waste of resources. Increasing communication and further explaining the project’s design and setting to participants, by organizing more briefings and meetings or using phone contacts, should help to improve the retention rate.

Although the CBPR project is a new approach in program planning for service delivery, our study showed that it is feasible and has benefits for promoting the family 3Hs. The approach is fully accepted by community partners and government district social welfare offices. The low dose and simple intervention design used in our programs are applicable to regular services/delivery and can be promoted on a territory-wide scale. Thus, incorporating positive psychology into the conceptual framework of service programs deserves further investigation. Better communication among working partners and exploration of the supporting social networks would be beneficial to the implementation of future projects. Our HFK project also has implications for policy in terms of resource allocation to districts, especially for areas with individuals of low socioeconomic status.

### Conclusion

In summary, this project was an innovative large-scale CBPR program, with implications for both academic and social services. Preliminary effective outcomes suggested successful implementation of the CBPR project, although this is subject to outcome analysis. This project combined the concepts of “best science” from academia and “best practice” from social services. In future programs, community workers should begin by adopting “best science” in development and evaluation, and enhancing awareness to maintain and sustain the program’s impact.

## Abbreviations

3Hs: health, happiness, and harmony
CBPR: community-based participatory research
HFK: Happy Family Kitchen
HKCSS: Hong Kong Council of Social Service
HKU: Hong Kong University
IRB: institutional review board
NGO: nongovernmental organization
SES: socioeconomic status
